# Typification of the genus *Dileptus* Dujardin, 1841 (Ciliophora, Rhynchostomatia)

**DOI:** 10.1016/j.ejop.2013.12.006

**Published:** 2014-06

**Authors:** Helmut Berger, Wilhelm Foissner

**Affiliations:** a Consulting Engineering Office for Ecology, Radetzkystrasse 10, 5020 Salzburg, Austria; b University of Salzburg, FB Organismische Biologie, Hellbrunnerstrasse 34, 5020 Salzburg, Austria

**Keywords:** *Dileptus anser*, *Dileptus margaritifer*, Nomenclature, Pritchard (1852), *Pseudomonilicaryon*

## Abstract

In their monograph of the dileptids, Vďačný and Foissner (2012) could not clarify the type species of the genus *Dileptus*Dujardin, 1841. Thus, they suggested that the problem be referred to the International Commission on Zoological Nomenclature. However, recently we discovered that Dujardin (1841) has originally typified *Dileptus* with *Amphileptus anser* sensu Ehrenberg (1838) which is in fact a misidentified *Amphileptus margaritifer*Ehrenberg, 1833, a common species also originally classified in *Dileptus*. Under Article 70.3.2 of the Code, *Dileptus margaritifer* (Ehrenberg, 1833) Dujardin, 1841, thoroughly redescribed by Foissner et al. (1995), is now the type of *Dileptus*. This has the great advantages of historical continuity and that new combinations (names) are not required.

## Introduction

The type concept caused great progress in the nomenclature of organisms (for a review, see Richter 1948). According to the International Code of Zoological Nomenclature (ICZN 1999, Article 61), each nominal taxon in the family, genus or species group has actually or potentially a name-bearing type. This provides the objective standard of reference for the scientific name. However, typification is sometimes difficult, especially for long-known taxa, e.g., for the ciliate genus *Dileptus* established by Dujardin (1841). This is only one of many unsolved cases as noted by Aescht, 2001, Aescht, 2008, Berger, 1999, Berger, 2001, and Foissner (2002).

Vďačný and Foissner (2012, p. 266) described the type species problem in *Dileptus* as follows: “Dujardin (1841) established the genus *Dileptus* with three nominal species: *Dileptus anser* (a misidentified *D. margaritifer*), *D. folium* (now *Litonotus cygnus*), and “*Dileptus* (*Amphileptus margaritifer*, Ehr. Infus. Pl XXXVII, fig. 5: 355)”, adopting the description from Ehrenberg (1838). He did not fix a type species. This was done by Fromentel (1875), using *D. folium*. Kahl (1931) overlooked Fromentel's typification and synonymized *D. folium* with *Litonotus cygnus*. Further, in his characterization of *Dileptus* on page 205, Kahl (1931) stated “typical species: *D. anser*”. Dragesco (1963) and Jankowski (1967) followed. However, under the Code, *D. anser* cannot be considered as type species of *Dileptus* because (i) the first author who subsequently designates one of the originally included nominal species validly designates the type species of that genus or subgenus (type by subsequent designation), and no later designation is valid (Article 69.1 of the ICZN 1999), and (ii) the term “designation” in relation to fixation of a type species [Arts. 68, 69] must be rigidly construed (Article 67.5 of the ICZN 1999). Thus, *D. folium* is the validly fixed type species of *Dileptus*, according to Articles 67.1.2 and 69.1 of the ICZN (1999). Unfortunately, *D. folium* is a junior synonym of *Litonotus cygnus*, a pleurostomatid ciliate belonging now to a different subclass, Haptoria (Vďačný et al. 2011). Thus, recognition of Fromentel's forgotten typification would cause changes in many well established ciliate names. Therefore, we shall bid the International Commission on Zoological Nomenclature to use its plenary power (i) to suppress Fromentel's (1875) typification of *Dileptus*, and (ii) to fix *D. margaritifer* as the type species of *Dileptus* because it is a well-known species (see description below), matching Jankowski's characterization of *Dileptus* and having slides deposited in an international repository”.

This exhaustive description of the type species problem demonstrates the complex situation in *Dileptus*. Fortunately, we discovered – by the studies of the little known books by Pritchard, 1852, Pritchard, 1861 – that Dujardin (1841) has fixed the type of *Dileptus* by original designation. Here we explain the history of *Dileptus* and its type species in the light of these “new” findings.

## Results and Discussion

### Brief history of *Dileptus* and *Dileptus margaritifer*

To explain the complex situation, we provide synonymies for *Dileptus* and its type species. The lists contain only entries, which are important for the explanation of the type species problem (see below). For more detailed lists, improved diagnoses, derivation of names, a comment on type and voucher material, and a comprehensive description, see Vďačný and Foissner (2012).

### *Dileptus* Dujardin, 1841

**Table:** 

1841	***Dileptus*** – Dujardin, Zoophytes, p. 404, 484 (original description, including type fixation). Type species (by original designation on p. 484): *Dileptus margaritifer* (Ehrenberg, 1833) Dujardin, 1841 (basionym *Amphileptus margaritifer* Ehrenberg, 1833). For detailed explanation, see below.
1852	***Dileptus*** – Pritchard, History of Infusorial Animalcules, p. 587, 591 (revision; note on type fixation by Dujardin 1841).
1861	***Dileptus*** – Pritchard, History of Infusoria, p. 636, 638, 639 (revision; note on type fixation by Dujardin 1841).
1875	***Dileptus*** – Fromentel, Études Microzoaires, p. 176, 177 (revision; invalid fixation of *Dileptus folium* Dujardin, 1841 as type species of *Dileptus* by subsequent designation).
1931	***Dileptus*****Dujardin, 1841** – Kahl, Tierwelt Dtl., 21: 204 (revision; mentions *Dileptus anser* (Müller, 1773) Dujardin, 1841 as type species of *Dileptus*).
1963	***Dileptus*****Dujardin, 1841** – Dragesco, Bull. biol. Fr. Belg., 97: 103 (revision; mentions *Dileptus anser* (Müller, 1773) Dujardin, 1841 as type species of *Dileptus*).
1967	***Dileptus*****Duj., 1840** – Jankowski, Mater. IV Konf. uč. Sekc. zool., year 1967: 36 (split of genus; mentions *Dileptus anser* (Müller, 1773) Dujardin, 1841 as type species of *Dileptus*).
2012	***Dileptus*****Dujardin, 1841** – Vďačný and Foissner, Denisia, 31: 265 (detailed revision; suggest to fix *A. margaritifer* Ehrenberg, 1833 as type species of *Dileptus* under the plenary power of the International Commission on Zoological Nomenclature).

### *Dileptus margaritifer* (Ehrenberg, 1833) Dujardin, 1841

**Table:** 

1833	***Amphileptus margaritifer*** – Ehrenberg, Abh. dt. Akad. Wiss. Berl., year 1833: 230 (original description without illustration).
1838	***Amphileptus anser*** – Ehrenberg, Infusionthierchen, p. 355, Tafel XXXVII, Fig. IV (Figs 7–10 in present paper; misidentification; used as type of *Dileptus* by Dujardin 1841).
1838	***Amphileptus margaritifer*** – Ehrenberg, Infusionsthierchen, p. 355, Tafel XXXVII, Fig. V (Figs 1–6 in present paper; revision and first illustration).
1841	***Dileptus Amphileptus margaritifer*****, Ehr.** – Dujardin, Zoophytes, p. 404 (combination with *Dileptus*).
1841	… **séparer des Amphileptes de M. Ehrenberg, son*****A. anser*****pour en faire le type de notre genre Dilepte (Voyez pag. 404–409)** – Dujardin, Zoophytes, p. 484 (original designation of *Amphileptus anser* sensu Ehrenberg as type of *Dileptus*; for detailed explanation, see below).
1931	***Dileptus (Vibrio) anser*****(O.F. Müller, 1786)** – Kahl, Tierwelt Dt., 21: 205 (revision, misidentification).
1984	***Dileptus margaritifer*****Ehrenberg, 1838** – Wirnsberger, Foissner and Adam, Arch. Protistenk., 128: 314 (incorrect authorship; comparison with *Dileptus anser*, now *Pseudomonilicaryon anser* (Müller, 1773)).
1995	***Dileptus margaritifer*****(Ehrenberg, 1833) Dujardin, 1841** – Foissner, Berger, Blatterer and Kohmann, Informationsberichte des Bayer. Landesamtes für Wasserwirtschaft, 1/95: 185 (ecological and morphological monograph and detailed description of African population; deposition of voucher material).
2012	***Dileptus margaritifer*****(Ehrenberg, 1833) Dujardin, 1841** – Vďačný and Foissner, Denisia, 31: 292, Fig. 91a–r, 92a–z, 93a–k, 94a–z, 95a–w (Figs 1–6 in present paper; detailed revision; suggest to fix *D. margaritifer* as type species of *Dileptus*).
2012	***Pseudomonilicaryon anser*****(Mueller, 1773) nov. comb.** – Vďačný and Foissner, Denisia, 31: 359, pro parte, Fig. 112a–d, **not** Fig. 111a–t, 112e–v, 113a–r, 114a–g (Figs 7–10 in present paper; assigned, obviously par lapsus, *Amphileptus anser* sensu Ehrenberg to *Pseudomonilicaryon anser*; see below).

Up to now, it was generally assumed that Dujardin (1841) established *Dileptus* without fixing a type species (see “Introduction”). To eradicate this flaw, three species have been proposed as type (basionyms given), namely *Dileptus folium* Dujardin, 1841 by Fromentel (1875), *Vibrio anser* Müller, 1773 by Kahl (1931), and *Amphileptus margaritifer* Ehrenberg, 1833 by Vďačný and Foissner (2012).

**Figs 1–14:**
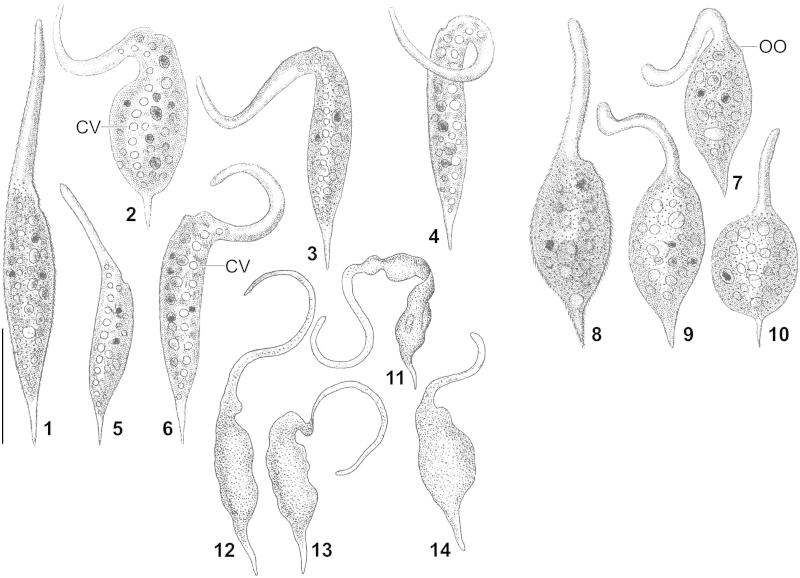
*Dileptus margaritifer* (1–6) and *D. anser* (7–10) according to Ehrenberg (1838) and *D. anser* (11–14) according to Müller (1786). Scale bar 100 μm. CV – contractile vacuoles, OO – oral opening.

**Locating the original designation of the type species in****Dujardin (1841)**. During the studies of Pritchard, 1852, Pritchard, 1861 little-known reviews, we recently discovered that Dujardin has made a typification: “The *Amphileptus anser* is taken by Dujardin as the type of a genus termed *Dileptus*, and *A. meleagris* of a genus *Loxophyllum*.” and “The type of this genus *Dileptus* is the *Amphileptus anser* of Ehrenberg; and the *A. margaritifer* (Ehr.) is referable also to it” (Pritchard 1852, p. 587, 591). Unfortunately, Dujardin (1841) did not designate the type (*Amphileptus anser* sensu Ehrenberg) in the *Dileptus* section on pages 404–410, but in the *Amphileptus* section on page 484 (see list of synonyms of *D. margaritifer*). Likely for that reason, all later workers, except for Pritchard, overlooked the type fixation. In spite of that, *Amphileptus anser* sensu Ehrenberg (1838) is type of *Dileptus* by original designation, and all later fixations are invalid (ICZN 1999, Article 70.2).

***Amphileptus anser*****sensu Ehrenberg (1838) is synonymous with*****Dileptus margaritifer*****(****Ehrenberg, 1833****) Dujardin, 1841**. *Dileptus anser* (Müller, 1773) Dujardin, 1841 – basionym *Vibrio anser*, now *Pseudomonilicaryon anser* (Müller, 1773) Vďačný and Foissner, 2012 – is a highly characteristic species with a very long and highly motile proboscis already described and illustrated by Müller, 1773, Müller, 1786, Figs 11–14; for monographic treatment, see Vďačný and Foissner 2012, p. 359). It is clearly different from the *Amphileptus anser* population described and illustrated by Ehrenberg (1838, Figs 7–10), which is very likely synonymous with *D. margaritifer*, as already proposed by Kahl (1931), Dragesco (1963), and Wirnsberger et al. (1984). By mistake, Vďačný and Foissner (2012, p. 359, 363) assigned *A. anser* sensu Ehrenberg (1838, Figs 7–10) to *Pseudomonilicaryon anser* (see list of synonyms). In the absence of type and voucher material, the identifications by Ehrenberg (1838) cannot be verified, but there is clear evidence that Ehrenberg's *Amphileptus margaritifer* (Figs 1–6) and *A. anser* (Figs 7–10) are identical.

The discussion demonstrates that Dujardin (1841) fixed a misidentified species as type of *Dileptus*, suggesting that Article 70.3 of the ICZN (1999) can be applied and *Dileptus margaritifer* can be chosen as type of *Dileptus*.

**Application of Article 70.3 of the****ICZN (1999)**. Article 70.3 of the Code states: “If an author discovers that a type species was misidentified, the author may select, and thereby fix as type species, the species that will, in his or her judgement, best serve stability and universality, either 70.3.1. the nominal species previously cited as type species, or 70.3.2. the taxonomic species actually involved in the misidentification. If the latter choice is made, the author must refer to this Article and cite together both the name previously cited as type species and the name of the species selected.”

This means that we can select between *Vibrio anser* Müller, 1773 (Article 70.3.1) and *Amphileptus margaritifer* Ehrenberg, 1833 (Article 70.3.2) because both species were originally included in *Dileptus* Dujardin, 1841 (Article 67.2).

With reference to Article 70.3.2 of the Code, the type species of *Dileptus* Dujardin, 1841 is now fixed as *Dileptus margaritifer* (Ehrenberg, 1833) Dujardin, 1841 (basionym *Amphileptus margaritifer*); in the original description of *Dileptus*, Dujardin (1841) fixed the synonymous *Amphileptus anser* sensu Ehrenberg (1838) as type species, a misidentified population as already proposed by previous monographers (Dragesco, 1963, Kahl, 1931, Wirnsberger et al., 1984). Of course, *Dileptus margaritifer* is also the type species of the nominotypical subgenus *Dileptus (Dileptus)* Dujardin, 1841 (Jankowski 1967).

We apply Article 70.3.2 because this serves best the spirit of the Code, i.e., stability and universality especially because no name changes are required. Further, *D. margaritifer* is well known and voucher slides have been deposited in a renowned repository, the Biologiezentrum of the Oberösterreichischen Landesmuseum in Linz (LI), Upper Austria (Aescht, 2008, Vďačný and Foissner, 2012).
